# Supplementary material for: Are carbon emissions trading and green financial instruments synergistic? -Comprehensive quantitative research based on content analysis
Source: PLoS One. 2024 Mar 7;19(3):e0298601. doi: 10.1371/journal.pone.0298601 (PMC10919601; doi:10.1371/journal.pone.0298601)
Supplement: S1 Data — (ZIP) [file pone.0298601.s001.zip › Data/地级市Z_F工作报告.zip!/╔╜╬≈/┤≤═1⁄4╩╨/└┤╫╘╣1⁄2╓┌║┼í╛┬φ┐╦╩2╛▌═°í┐_2016.docx]

各位代表：

现在，我代表市人民政府向大会报告工作，请予审议，并请市政协委员和其他列席人员提出意见。

一、2015年和“十二五”时期工作回顾

2015年是我市发展历程中极不平凡的一年。面对严峻复杂的经济形势和艰巨繁重的改革发展稳定任务，全市上下认真贯彻落实党的十八大，十八届三中、四中、五中全会精神和习近平总书记系列重要讲话精神，在省委省政府和市委的正确领导下，凝心聚力，攻坚克难，较好地完成了主要工作目标，实现了经济社会持续平稳发展，开启了建设美丽大同、富裕大同、幸福大同的新征程。

一年来，主要做了以下工作：

（一）积极作为稳增长。全市地区生产总值达到1052．9亿元，增长9％；规模以上工业增加值增长8．4％，全社会固定资产投资增长6．8％，社会消费品零售总额增长4．8％；城镇常住居民人均可支配收入24771元，增长7．5％；农村常住居民人均可支配收入7708元，增长8％。地区生产总值增速、规模以上工业增加值增速、二级以上优良天数和空气质量综合指数四项指标居全省第一。但受经济下行和结构性减税等多重因素影响，公共财政预算收入出现多年来首次负增长，比上年下降12％。把千方百计稳增长作为首位工作来抓。制定并实施56条稳增长措施，为企业减负52亿元。推进大用户直供电，使7户用电企业减少电费4400多万元，3户发电企业增加产值2亿元。全力支持大企业发展，以同煤集团为主的省属企业拉动全市工业增长13．8个百分点。下大力气解决融资瓶颈问题。采取“一对一”精准帮扶，为296户中小微企业协调信贷资金48亿元。扩大“营改增”试点，2403户商企受惠。发挥农业示范园区带动作用，建成设施农业、标准化养殖和农产品加工园区30个，农产品加工龙头企业实现销售收入77．8亿元。统筹规划，制定政策，大力促进文化旅游、电子商务等新业态发展。电子商务园区投入运营，注册的电商企业达1243家。旅游总收入达到281．2亿元，增长17．8％。

（二）加大力度调结构。坚持把项目投资作为调结构的重要抓手，完成全社会固定资产投资1145．4亿元。实施省市重点项目940个、工业调产项目236个。非煤产业投资和民间投资占比分别达76％和63．8％。签订招商引资项目264个、总投资1711．4亿元。总装机100万千瓦的国家光伏示范基地项目全部开工建设，同煤塔山二期、同煤阳高、京能左云3个在建低热值煤发电项目进展顺利，同煤浑源2×35万千瓦热电联产、同煤大唐三期1×66万千瓦热电联产、国电湖东2×100万千瓦发电项目取得路条。中海油煤制气、同煤烯烃项目环评获批。全市人民热切期盼的大张高铁开工建设，大西高铁前期工作进展良好，大乌高铁列入铁总“十三五”规划。

（三）锐意创新添活力。关闭破产企业4户，安置职工729人。实行市属国有企业重大信息全面公开。大同商业银行改制为大同银行，新增村镇银行2家、“新三板”挂牌企业1家、Q板挂牌企业2家。争取地方政府债券60亿元。中国国际技术转移中心大同工作站、晋投玄武岩院士工作站挂牌成立，7家高新技术企业通过认定。全面推行“三证合一”、“一照一码”，新增市场主体2．5万户。深化户籍制度改革，新增城镇户籍人口1．3万人。调整管理使用制度，释放住房公积金41．5亿元。医药、卫生、教育等改革稳步推进，农村土地确权登记颁证试点工作进展良好。党政机关公车改革基本完成。成功举办第二届乌大张联席会议。左云经济技术开发区获批为省级开发区。

（四）坚持不懈搞建设。着眼于统筹提质和联动联创，继续狠抓城乡基础设施建设和人居环境改善。新建续建城市道路48．1公里。推进城市管网工程，城市供水普及率、集中供热普及率和气化率分别达到99．8％、99．7％和98．6％。北环桥、客运东站主体完工。全速推进制约城墙合拢的拆除、搬迁和建设工程。代王府中轴线、明堂公园修复工程主体完工。灵涞高速公路建成通车，京新高速公路天镇段主体完工，国道108线改建工程开工建设。改造县乡公路183．5公里，完成农村广播电视卫星户户通5万户，新建农村老年人日间照料中心75个。

（五）多措并举抓生态。深入开展大气污染防治，努力创建国家环保模范城市。完成淘汰黄标车和更新公交车工作。扎实推进水污染防治行动计划，扩大餐厨垃圾、污泥资源化处理能力，八条主要河流断面水质基本稳定。狠抓重点领域节能减排，万元GDP综合能耗下降5．5％，工业固废综合利用率65．2％。实施重点生态建设工程，完成营造林32万亩。完成南城墙景观绿化、御东公共活动走廊建设和儿童公园改造一期工程，建成区绿化覆盖率、绿地率分别达40．1％、36％，人均公园绿地面积达14．8平方米。

（六）综合施策惠民生。在经济下行和财政困难的情况下，改善民生的力度没有减弱，投入的资金没有减少。全年用于保障和改善民生的资金达252．6亿元，占财政支出总额的86．9％。城镇新增就业5．6万人，农村劳动力转移就业3万人，减少贫困人口5．9万人。城乡低保、养老保险等提标工作全面完成。推动义务教育均衡发展，继续改善学校办学条件，各类校舍建设任务全部完成。全面启动分级诊疗制度，实现市县两级组建医疗联合体工作全覆盖，基本消除了卫生室空白村。开工建设保障性住房7．9万套，基本建成1．6万套。投资36．6亿元改善农村人居环境。高度重视安全生产工作，事故起数和死亡人数实现双下降。扎实推进平安大同建设，严厉打击各类违法犯罪，积极化解各种利益诉求，促进了社会和谐稳定。

（七）政府建设重实效。自觉接受人大法律监督和政协民主监督，认真听取社会各方面意见建议，113件人大代表建议和238件政协委员提案全部办复。认真落实“六权治本”要求，率先在全省公布市级政府部门权力清单和责任清单，取消、调整、下放行政审批等权力事项374项，清理行政职权3747项，精减率56．9％。强化综合协调，强力攻克项目手续办理难、工程竣工验收难，深度优化行政审批和政务服务。坚决执行中央八项规定，坚决反对“四风”，坚决落实“两个责任”。扎实推进依法治市，依法行政水平进一步提高。深入开展“三严三实”专题教育和学习讨论落实活动，政府系统干部作风持续好转。积极开展“同心同力发展大同”大调研大讨论活动，有力地促进了政府工作的科学化和民主化。

2015年入冬以来，针对我市项目冬眠、工程冬休、工作冬闲的惯性特征，市委市政府以加快发展闲不起、推动转型等不起、振兴大同输不起的使命担当，在全省率先发起“冬季行动”攻坚战，得到了省委省政府充分肯定和大力推广。在五个月的时间里，我们党政齐上阵、干群共奋进，马上就办、真抓实干，取得了丰硕成果。完成的征收量相当于之前两年的总量，使80％的待安置户居有定所；手续办理时限平均缩短79％，办结的审批事项是上年同时段的2．2倍；招商引资签约总投资是预定目标的3．6倍，10亿元以上项目达32个；加快推进11项金融创新举措，融资百亿的目标基本完成；市直部门办结帮扶企业事项148项，县区办结222项；省交办的269件信访案件全部化解，40件重点集体信访案件处置到位。“冬季行动”为今年乃至整个“十三五”的工作开好局起好步，争取了时间、赢得了主动、凝聚了力量。

各位代表，总结2015年，困难比预料的多，结果比预想的好。回顾整个“十二五”的发展变化，尽管遇到了经济上、政治上等多方面的“立体式困扰”，特别是经历了煤炭行业深度调整的阵痛，但我们最终顶住了压力，战胜了挫折，创造了辉煌。

——这五年，是大同综合实力明显增强的五年。地区生产总值年均增长9．4％，总量由2010年全省第9上升到2015年第5；公共财政预算收入年均增长10．9％，总量由2010年全省第8上升到2015年第5。累计新开工项目3447个，完成固定资产投资4750．7亿元，是“十一五”时期的2．9倍。

——这五年，是大同经济结构加速调整的五年。三次产业比由2010年的5．2∶48．7∶46．1调整为2015年的5．3∶41．8∶52．9，三产超二产11．1个百分点。服务业增加值增长1．7倍，旅游总收入增长2．1倍。

——这五年，是大同城乡面貌深刻变化的五年。建成区面积达到134．2平方公里，是2010年的1．4倍。御东新区迅速崛起，古城内重点文物保护与修复基本完成。新增城市道路面积683万平方米、绿化面积1402万平方米。安置城市和工矿棚户区居民20．3万户，农村危房改造10．5万户，抗震房改建2．1万户，解决了34．1万人饮水安全。实现高速公路县县通、水泥路（油路）村村通，行政村街道全部硬化亮化。城镇化率提高6．1个百分点，达到61％。森林覆盖率由2010年的20．1％上升到2015年的23．5％。

——这五年，是大同知名度显著提升的五年。先后获得国家园林城市、国家新能源示范城市、全国性综合交通枢纽城市、中国十佳运动休闲城市、最具生态竞争力城市、区域流通节点城市等荣誉称号，实现了全国双拥模范城“七连冠”。成功举办了世界养生大会、国际太阳能大赛、国际自行车骑游大会、国际雕塑壁画双年展、云冈文化旅游节等大型赛事和节庆活动。云冈石窟、北岳恒山、大同古城等旅游文化景点景区的品牌效应不断放大，大同的知名度和影响力大幅提升。

——这五年，是大同人民群众得到实惠最多的五年。2015年城乡居民人均可支配收入分别是2010年的1．5倍、1．9倍，年均分别增长9％、13．7％。企业离退休人员基本养老金人均待遇由2010年的1350元提高到2504元，城市低保标准由2010年的256元/月提高到最高449元/月，农村低保标准由2010年的1080元/年提高到2658元/年。21．1万农村贫困人口脱贫，贫困发生率从2010年的18％下降到2015年的12％。

各位代表，“十二五”时期，在省委省政府和市委的正确领导下，在市人大、市政协的监督支持下，全市上下勇于创新、知难而进，大同的发展取得了有目共睹的历史性成就。在此，我代表市人民政府，向全市人民，向驻同解放军、武警官兵、公安民警和中央、省属驻同单位，向尽心履职的各位人大代表、政协委员，向所有关心、支持、参与大同建设发展的社会各界朋友，表示崇高的敬意和衷心的感谢！

各位代表，回顾过去，成绩令人鼓舞；居安思危，形势不容乐观。我们清醒地认识到，我市经济社会发展中还面临着诸多深层次的矛盾、困难和问题。主要是：经济总量不大，结构不优，质量效益不高的矛盾仍然突出，“一煤独大”的格局没有改变；传统产业产能过剩，新兴产业短期内难以形成有效支撑，去产能、去库存、去杠杆、降成本、补短板的任务极为繁重；公共资源难以满足不断增长的群众需求，民生保障能力相对较弱，脱贫攻坚任务大，共享发展的水平亟待提高；对外开放的动能不足，创新资源集聚能力不强，招商引资精准性、有效性亟待增强；廉洁和安全理念还没有深入人心，全社会重视安全的大环境还没有形成，慢作为、不作为、乱作为问题比较突出，“四风”和腐败问题还时有发生。从近期看，煤炭价格止跌回升乏力，产能压减力度不断增大，致使占主体地位的煤炭行业产销量和效益指标呈急速下滑之势，稳增长、保收入面临着更为严峻的挑战。病之所在，治之所要；民之所望，施政所向。我们将牢记责任使命，进一步增强忧患意识和担当意识，扎实有效解决问题，绝不辜负人民厚望。

二、“十三五”时期总体发展思路和主要目标任务

“十三五”时期是我国全面建成小康社会的决胜阶段，也是我们同心同力发展大同的关键时期。我们既面临着挑战，也面临着机遇。最大的挑战是世界经济总体低迷、增长乏力，我国经济的外部需求尤其是能源需求持续减弱，经济下行压力有增无减。但我国经济发展长期向好的基本面没有改变，仍然处于大有可为的战略机遇期。经济发展步入新常态，新一轮科技革命和产业变革蓄势待发，“一带一路”战略深入实施，科技创新成为主流主导，供给侧结构性改革强力推进，结构性减税持续发力，投融资体制改革不断深化，社会保险缴费率大幅降低。这既是必须顺应的大趋势，也是不断释放的大机遇。更应该看到，融入京津冀、对接环渤海、参与乌大张等国家级区域发展战略体系，大张、大西、大乌高铁的陆续建成通车，将更加凸显我市独有的地缘优势和比较优势，成为我们加快发展速度、提升发展级次的长期利好因素。我们要审时度势、主动担当，知难而进、锲而不舍，奋力开创出大同发展的新局面。

根据市委十四届七次全体会议精神，“十三五”时期，我市经济社会发展的指导思想是：高举中国特色社会主义伟大旗帜，全面贯彻党的十八大和十八届三中、四中、五中全会精神，坚持以邓小平理论、“三个代表”重要思想、科学发展观为指导，深入贯彻习近平总书记系列讲话精神，按照中央“四个全面”战略布局和省委“五句话”总要求，牢固树立创新、协调、绿色、开放、共享、廉洁和安全发展新理念，以转型综改试验区建设为统领，以转方式、调结构、增效益、提速度为主线，紧紧围绕打造东承首都、西接丝路、南贯三晋、北通蒙俄“区域性中心城市”的基本定位，着力加快转型发展，着力推进改革创新，着力满足群众需要，着力强化涵养生态，着力维护安全稳定，着力加强从严治党，同心同力建设美丽大同、富裕大同、幸福大同，为全面建成小康社会、谱写好“中国梦”的大同篇章而努力奋斗。

围绕上述总体思路和发展战略，根据《中共大同市委关于制定国民经济和社会发展第十三个五年规划的建议》，市政府编制了《国民经济和社会发展第十三个五年规划纲要（草案）》，提交大会审议。

《规划纲要（草案）》提出，“十三五”时期我市经济社会发展的主要目标是：在提高发展平衡性、包容性、可持续性的基础上，实现“两中高”、“两翻番”、“两快于”、“两突破”。即努力争取经济总量迈入全省中高位，产业发展迈向行业中高端；确保地区生产总值比2010年翻一番，城乡常住居民人均可支配收入比2010年翻一番；农村常住居民人均可支配收入增长快于城镇居民，贫困地区农民收入增长快于全市居民；到2020年，新型工业化、信息化、城镇化、农业现代化建设取得重大突破，美丽大同、富裕大同、幸福大同建设取得重大突破，大同与全国、全省一道同步实现全面小康。

实现上述目标，主要途径和举措有以下六个方面。

一是推进创新发展，不断增强发展动力。立足科技创新，实施创新驱动和人才强市战略，建设御东互联网产业园、科技孵化中心、技术创新中心和创客公园，培育一批具有竞争力的创新型领军企业，大数据、云计算、物联网广泛使用，科技与经济深度融合，研发经费投入强度超过2．5％，科技进步对经济增长的贡献率突破60％。“革命兴煤”工程取得重大突破，新产业、新业态和新增长点快速生成，多元支柱的产业格局基本形成。

二是推进协调发展，着力优化发展布局。统筹推进区划调整和新型城镇化，加快推进“数字大同”和“智慧大同”建设，到2020年，常住人口城镇化率达到65％，户籍人口城镇化率达到58％。加速推进产城融合，城市的宜居性、城乡的和谐度、居民的幸福感显著增强。全力推进新农村建设，大力发展都市型现代农业，培育建设一批辐射带动能力强的乡镇群和增长极，城乡一体化发展格局基本形成。

三是推进绿色发展，倾心打造美丽家园。推进能源资源“双控”，加快主体功能区布局和生态安全屏障建设，促进低碳循环发展，基本形成绿色低碳发展方式和生活方式。“十三五”期间，森林覆盖率年均提高0．5个百分点，建成区绿化覆盖率年均提高1个百分点，实质性推进生态涵养示范区和“首都后花园”建设。

四是推进开放发展，大力拓展发展空间。主动对接“一带一路”，务实参与“中俄蒙经济走廊”建设，不断深化与京津冀三地合作，全力打造京津产业转移重要战略区域，加快融入环渤海合作发展圈，拓展跨区域发展的深度和广度。推动乌大张跨区域合作和晋陕蒙能源富集区合作。积极承接发达地区产业转移，努力构筑晋北区域合作和对外开放新高地。

五是推进共享发展，切实增进民生福祉。不断加大对民生工程和社会事业投入，健全就业、住房、社保、教育、医疗卫生等公共服务体系，努力让发展成果更多更公平更实在地惠及人民群众。基本消除区域性整体贫困，实现农村贫困人口稳定脱贫，贫困县全部摘帽。城乡常住居民人均可支配收入年均分别增长6．5％左右和6．5％以上，新增城镇就业20．5万人。

六是推进廉洁和安全发展，全面创优发展环境。扎实推进“六权治本”，从源头上织密编牢制度的笼子。坚持不懈抓好安全生产。建设更有保障的质量安全体系和更加完善的公共安全保障体系，初步形成全民共建共享的社会治理格局，实现廉洁安全发展与经济社会发展的良性互动。

各位代表，目标承载使命，任务考验担当。只要我们同心同力，苦干实干，宏伟蓝图就一定能够变成现实，全面建成小康社会的目标就一定能够如期实现！

三、2016年主要工作

2016年是实施“十三五”规划的开局之年，也是推进供给侧结构性改革的攻坚之年，做好今年的政府工作意义十分重大。

全年经济社会发展预期性目标是：地区生产总值增长6．5％以上，固定资产投资增长12％，社会消费品零售总额增长6％，公共财政预算收入下降6％，城镇常住居民人均可支配收入增长6．5％左右，农村常住居民人均可支配收入增长6．5％以上，新增城镇就业岗位4．1万个，城镇登记失业率控制在4．2％以内，居民消费价格涨幅控制在3％左右。全面完成省下达的安全生产和节能减排等约束性指标。

围绕实现上述目标，我们要重点抓好八方面工作：

（一）推动供给侧结构性改革，促进经济平稳健康增长。坚持稳定经济增长和推进结构性改革有机结合，优化存量、引导增量、主动减量。

积极稳妥去产能。严格控制新增产能，坚决淘汰落后产能，有序退出过剩产能。对资不抵债、扭亏无望的“僵尸企业”、“僵尸项目”，妥善处置，市场出清。通过“五个一批”，自觉压减煤炭过剩产能，三年内退出7座60万吨以下煤矿，压缩产能360万吨。支持同煤集团改革，实现转型升级。

千方百计降成本。坚决贯彻落实各项减轻企业负担的政策措施，进一步抓实简政放权、放管结合、优化服务工作，建立完善收费清单制度，清理规范涉企收费，降低企业制度性交易成本。积极引导企业创新经营，强化内部管理，削减非生产性支出，降低成本费用。统筹用好各类政府性基金和专项扶持资金，促进银企对接，缓解企业融资贵问题。

多种途径去库存。重点是打通商品房、安置房、保障房转换渠道，积极消化房地产库存。引导和鼓励房地产企业开发养老、文化、科教、体育等新型房地产项目。支持装配式建筑产业发展。建立购租并举的住房制度，提高棚户区改造货币化安置比例。扩大公租房服务对象，发展住房租赁市场，促进房地产市场健康发展。实现住房公积金缴存异地互认和转移接续，推进异地房贷业务。

坚定不移扩投资。扎实推进“项目创新年”活动，紧盯国家投资七大重点方向和省十大重点领域，做好项目谋划，加快前期工作，狠抓项目落地建设。组织实施七大板块632个投资项目，完成投资1300亿元以上。重点实施总投资140亿元的现代农业项目、540亿元的新型工业化项目、195亿元的现代服务业项目、85亿元的大交通项目、130亿元的城镇化建设项目、130亿元的生态建设项目和80亿元的民生保障改善项目。通过加强组织领导、加快项目实施、加大考核督查，让有效投资发挥稳增长、调结构、惠民生的最关键作用。

（二）构筑项目支撑体系，促进产业结构调整。以项目为抓手，切实优化传统产业、培育新兴产业、壮大接续产业，做好煤与非煤两篇大文章。

加快工业转型升级。推动煤炭产业集约清洁高效发展。坚持有保有压，认真落实产能置换政策，加快推进同煤3个千万吨矿井建设，严格控制产能总量。实施煤电一体化战略，抓好坑口电厂建设。塔山电厂二期项目并网发电，3个低热值煤电厂尽早完工，打造千万千瓦级煤电基地。下大力气延伸煤炭产业链，加快发展新型煤化工产业。重点推进煤制气、甲醇、烯烃等项目，打造晋北煤化工基地。充分发挥比较优势，培育壮大新兴产业。支持高速列车受电弓、玄武岩深加工、碳纤维汽车骨架等项目开发建设，稳妥开发石墨资源和石墨烯产业，加快发展新材料产业。以中车、山柴、大齿等大型企业为依托，扩大北宇新能源电动乘用车产能产量，推进瓦斯发电机组研制生产，做好轻轨地铁研发生产基地项目，推进通航产业园建设，打造装备制造和轨道交通基地。依托两个医药园区，推进威奇达光明药业重组、好医生中草药、浑源黄芪科技开发等项目建设，努力建成全球最大的抗生素生产基地。抓紧落实国家光伏示范基地二期100万千瓦项目，推进风力发电项目建设，加快浑源抽水蓄能调峰电站项目开工建设，争创智慧能源产业园区，打造千万千瓦级新能源基地。

做大做强文化旅游产业。围绕景区景点、线路设计、基础设施、服务提升、营销推广“五环联动”，坚持扩容、提质、升级、增效，加快体制机制创新，形成以古城为核心、东南西北四线同步发展的“一核四线”旅游发展格局。实施“互联网＋旅游”行动，推广旅游企业在线服务，加大旅游营销力度，促进旅游产业转型升级，建设旅游休闲度假目的地、山西旅游北大门和全省旅游集散中心。加大火山群、平型关、大泉山等景点景区建设力度。开通大同－北京、大同－太原旅游专列，启动沿长城旅游公路、永固陵至208国道旅游专线建设。推进房车营地度假项目。积极申办全省旅游发展大会。深度开发民间工艺品。充分发挥节庆和会展的带动作用。推进文化产业与旅游业深度融合。加快推进市县两级公共文化体育场馆建设。打造煤气工业遗址文化创意园。方特欢乐世界主题公园暑期开门迎客。

大力发展现代服务业。加快电子商务与物流协同发展试点城市建设工作，全面推行电子商务交易，新增10家电商企业。推进万昌物流二期、上海文通等项目建设，争取华阳玛里纳陆港、晋北国际物流园区等项目年内开工。全力打造现代养老产业，建设御东新区、大同县火山群康养基地，实施阳高温泉养生镇综合开发项目。注重发展绿色、时尚、品质消费。

（三）聚焦脱贫攻坚，统筹做好“三农”工作。始终把“三农”工作作为重中之重，把脱贫攻坚作为重要政治任务和第一民生工程，完善政策，落实责任，大力扶持，统筹推进。

扎实推进现代农业发展。完成高标准农田建设18万亩，推广测土配方施肥350万亩，实施膜下滴灌1．3万亩。新增设施农业1万亩，发展道地中药材种植基地2万亩。支持小杂粮产业做大做强。新建扩建标准化养殖小区200个，整合带动个体养殖发展。推进原料生产、加工物流、市场营销融合发展。加大“三品一标”认证力度，打造面向京津冀、面向环渤海、面向全国的特色农业品牌。不断深化与顺鑫、中地、首农等京企合作，积极推进正大生猪全产业链和内蒙古香岛光伏设施农业等总投资上百亿元的大项目。突出大园区建设，新建30个规模大、效益好、带动强的现代农业标准园区。

全面提升农村建设和社会治理水平。以改善农村人居环境工程为抓手，促进城市基础设施向农村延伸、公共服务向农村拓展、城市文明向农村辐射。加大贫困地区自然村和采煤沉陷区村庄搬迁力度，加大传统村落民居和历史文化名村名镇保护力度。创建10个省市级美丽宜居示范村和840个省级达标村。继续深化农村改革。完成48个乡镇、1000个村的农村土地确权登记颁证工作。扩大土地经营权抵押、担保试点，健全农村产权交易市场体系。

确保完成年度脱贫攻坚任务。积极开展贫困统计监测，加强贫困动态管理，实现扶贫台账与脱贫台账相衔接、相对应、可追溯，努力提高扶贫质量。以连片特困地区为主战场，把“五个一批”真正落实到村、到户、到人。对3．2万具有劳动能力的贫困人口，通过企业产业扶持和提供培训、金融支持等手段实现脱贫；对1．3万“一方水土养不起一方人”的贫困地区人口，分类引导群众向县城、重点镇和中心村搬迁脱贫；对1万名贫困户子女加大教育资助力度，阻断贫困代际传递；对0．5万丧失劳动能力以及符合低保、五保和残疾条件的贫困人口，通过光伏扶贫和社会保障政策兜底脱贫。认真执行脱贫攻坚目标、任务、资金、权责“四到县”制度，扎实开展驻村帮扶，深化市直部门对口帮扶，做到分工明确、责任清晰、任务到人、考核到位。构建扶贫资金多元投入机制，加大财政扶贫投入力度，引导更多金融资金和社会资本参与扶贫开发，形成专项扶贫、行业扶贫、社会扶贫“三位一体”的大扶贫格局，务必实现210个贫困村摘帽、6万贫困人口脱贫的目标。

各位代表，我们决不能忘记农民群众曾经作出的历史贡献和巨大牺牲，必须始终带着对农民群众的深厚情感，以“贫困不除、愧对历史”的使命感、“农民不富、寝食难安”的责任感，时刻把农民群众的幸福放在心上、抓在手上，决不能让一个贫困人口在全面建成小康社会的进程中掉队落伍。

（四）增强服务人民理念，加快新型城镇化步伐。按照中央“一个尊重、五个统筹”的要求，围绕建设区域性中心城市目标，切实转变规划建设管理理念，推动城镇化由建房为主向以人为本转变，由重城轻乡向城乡统筹转变；城市发展由外延式扩张向内涵式提升转变，由产城分置向产城融合转变；城市建设由以建为主向建管并重转变，由政府投资向多元融资转变。

提高城乡规划管理水平。按照“一主三副，一轴一带”市域空间布局，完善重点区位控制性详规，推进“多规合一”。高度重视城市设计。稳步推进城市综合执法，实现城市分级分层管理。持续推动城乡清洁工程，开展达标创建活动，创建省级保洁示范街、容貌示范街。加快云计算和数据中心建设，全面推广数字化城管模式。

推动城市内涵式发展。统筹协调老城区、御东区和口泉区发展，积极谋划实施城市快速道路和轨道交通，加快实现功能对接、高度融合、同步发展。加大古城保护力度。全面推进代王府片区的修复和开发，钟楼、太平楼主体建成，明堂公园基本完工，西城墙主体合拢，护城河全线贯通，南、西、北城墙带状公园和平城街拓改工程全部完工。积极推进古城整体开发，进一步完善古城管理体制，努力把古城打造成最有实力、最显活力、最具魅力的大产业，延续文脉，提升功能，集聚人气，激活助火古城，实现广大群众多年的夙愿。加快旧城改造步伐。新建续建城市道路工程154项，北环路拓改竣工，北环桥建成通车，开源桥力争主体完工，畅通微循环，打通断头路，建设便民路。加快御东区功能配套。搞好公共服务，有效承接产业、人口和城市功能转移。积极探索以市场化手段推进大剧院、美术馆、体育中心的建设运营，图书馆年内投入使用。加快万达商业综合体建设进度。围绕高铁站场建设打造高铁商务圈。让御东新区尽快成为宜居宜游宜业和山水林田湖特色市区。加快推进口泉中心区建设。统筹协调，同步规划，大力推进生态环境综合整治工程，完善基础设施配套和公共服务功能，建设低碳、绿色、集约、智慧的现代化市区。

持续改善城乡人居环境。扎实推进城市人居环境改善工程，征收安置1．7万户、改造棚户区5．5万户。建成污泥干化处理厂，启动融雪剂熔化池建设。坚持地上地下并重，道路管网同步实施，启动建设地下综合管廊。加强大县城和重点镇建设。以提升承载能力为目标，强化中心城区各组团与县城之间的产业协作协同，逐步形成横向错位发展、纵向分工协作的发展格局。继续推进农村人居环境改善工程，圆满完成2．9万户农村危房改造和抗震住房改建任务，启动9321户采煤沉陷区和农村地质灾害治理搬迁工作。

（五）不断强化求新图变意识，切实加大改革创新力度。面对发展竞争格局的深刻变化，顺应经济提质增效的迫切需求，强化改革推动、创新驱动、开放带动，增添改革新动力，增强发展新动能，增创开放新优势。

坚持深化重点领域改革，多措并举增强改革的协同性。狠抓2016年转型综改行动计划落实。深化国资国企改革，组建国有资本投资运营公司，完善政府资产管理运营办法，基本完成市属国有企业股份制改造和脱钩改革工作。深化扩权强县、扩权强镇改革，扩大县域经济发展的自主权。健全县域产业合作发展和利益分享机制，鼓励县区共建产业合作园区，共同培育区域公共品牌和地理标志保护产品。创新开发区体制机制。完善林光互补一体化发展机制，保障光伏产业用地。优化集团校、联盟校办学机制。稳步实施行政区划、财政税收、不动产统一登记、国有林场、供销社等项改革。

强力推动三大突破，多层联动激发经济的创新力。推进金融创新。做大做实政府产业发展引导基金，组建旅游文化产业发展基金，增发定向企业债券。推进华晟果蔬、华林商贸、华泰餐饮等7家企业挂牌工作。完成大同农村商业银行和市级融资租赁公司组建。启动金融街二期工程。全面改革政府投融资和建设开发模式，大力推广PPP，积极争取国家专项建设基金等长期限、低成本资金的流入。支持扩张保险险种、扩展担保公司担保续贷能力。探索设立民间资本投资服务中心、民间资本管理公司。推进科技创新。打造“创业苗圃—孵化器—科技园—科技城”链条。深化京同科技创新战略合作，争取建设大同中关村科技创新大楼，共建科技创新基地，承接科技产业转移。启动大同大学科技园创建工作。新发展高新技术企业5家，新建省级工程技术研究中心1个。力推众创、众扶、众包、众筹等新模式，打造众创空间5个。大力发展民营经济。全面贯彻落实促进民营经济和中小微企业发展的各项扶持政策，放宽准入条件，搞好跟踪服务，打造政府与民营企业良性互动的服务平台，推动全领域、全产业链向民间资本开放。

积极推动对外合作交流，多方出击提升市域的外向度。继续培育出口企业，扩大出口规模。积极发展蒙俄国际贸易和投资合作，重视参与国际产能合作。深化乌大张三市在基础设施、生态共治、文化旅游等重点领域的合作。全力落实对接京津冀行动计划，务求不断取得实效。广泛参加和全力办好各种引资引才引智活动。推动航空口岸正式开放，争取陆路口岸开放，增开国际国内客运航线，强化全国性综合交通枢纽城市地位，着力建设人尽其才、物尽其流、资尽其用的开放发展高地。

（六）持续推进综合治理，不断改善生态环境。牢固确立绿水青山就是金山银山的理念，始终把生态建设和环境保护融入经济社会发展的全过程，务必守住资源消耗上限、环境质量底线、生态保护红线。

提高资源利用水平。促进煤矸石、粉煤灰、脱硫石膏等大宗工业固废综合利用，加强废旧家电分类回收和再生资源回收利用。提高建筑节能标准，发展绿色建材，推广绿色建筑，完成现有住宅节能改造100万平方米。坚持公交优先，推动绿色出行。深入开展节能、节地、节材、节水活动，争创节约型机关、节约型企业。

加大环境保护力度。坚持源头严防、过程严管、后果严惩。抓紧完成新增供热能力1000万平方米的机组改造工程，加快燃煤锅炉升级改造，取缔10蒸吨以下燃煤锅炉。加强秸秆焚烧火点监控和扬尘污染综合防控。完成淘汰黄标车和老旧车任务。确保空气质量二级以上优良天数稳中有升。全面落实水污染防治行动计划，促进水质稳步提升。完成浑源县、大同县污水处理厂提标改造以及新荣区、大同县生活垃圾转运站和天镇县生活垃圾无害化处理场建设。超前筹划土壤污染防治行动，适时开展土壤环境监测。探索推行环境污染第三方治理。

加快生态建设进度。按照“三加三不减”的要求，抓好京津风沙源治理、退耕还林还草等重点工程建设，完成营造林32．5万亩。突出抓好生态保护与修复工作，抓紧组织实施御河、口泉河流域生态综合治理重大项目，积极谋划洋河、浑河、唐河等流域生态综合整治工程，开工建设明堂遗址公园景观、大同公园改造等项目。

（七）更加关注民生保障改善，全力维护社会和谐稳定。越是困难的时候，越要守住底线、突出重点，多谋民生之利，多解民生之忧。

扎实做好就业创业和社会保障工作。实施更加积极的就业政策，重点解决好零就业家庭、高校毕业生、复转军人、城镇困难人员等特殊群体就业工作，鼓励农民就地转移就业和外出务工人员返乡创业。推进医保、医疗、医药三医联动，实施全民参保登记计划，推动社会保障由制度全覆盖到人群全覆盖。全面实施困难残疾人生活补贴和重度残疾人护理补贴制度。落实经济困难的高龄与失能老年人补贴以及百岁以上老年人补贴新标准。继续提高城乡低保标准，推动农村低保标准与国家扶贫标准相衔接。推进医疗救助“一站式”即时结算服务，完善重特大疾病医疗救助政策。

努力提高城乡居民收入。依法推进企业工资集体协商，落实最低工资标准政策。完善机关事业单位工资制度，落实县以下机关公务员职务与职级并行制度和乡镇工作补贴。不折不扣地执行好各项强农惠农政策，拓宽农民增收渠道，夯实农民增收基础。

协调发展各项社会事业。办人民满意的教育。四个县区义务教育均衡达标通过验收，新改扩建农村幼儿园28所，加快新建小区义务教育学校配套建设，完成特教学校、二十一中等学校校舍或设施建设工程。加强职业教育市级统筹，整合资源，完善办学体系，加快培养实用技术人才。支持和规范民办教育发展。加快高等教育发展步伐。巩固扩大县级公立医院综合改革成果。全面实施两孩政策。完成市中心血站迁建、全科医生培养基地项目建设。加大社区建设力度，实行居民健康卡与其他公共服务卡一卡通用。落实全民健身计划，构建全民健身服务体系，提高人民健康水平。加强国防教育和国防后备力量建设，持续开展双拥工作，推进军民深度融合发展，巩固和发展军政军民团结。支持工会、共青团、妇联等人民团体更好地发挥桥梁纽带作用。做好外事、侨务、港澳、对台工作。加强民族宗教、参事、史志、文史、档案、测绘、防震减灾、对口援疆等工作。

创新社会治理。加大领导下访接访力度，强化信访责任落实，实行党政联席会议制度，共同化解处理信访积案，依法维护信访秩序，坚决遏制群体性上访势头。加强社会治安综合治理，强化应急管理体系建设，推进立体化社会治安防控体系建设，重拳打击各类违法犯罪活动。按照“党政同责、一岗双责、失职追责”的要求，落实各级政府安全生产监管责任，强化企业安全生产主体责任，采取“四不两直”方式，突出煤矿、非煤矿山、危险化学品、道路交通、消防安全等重点行业领域，加大隐患排查和打非治违专项整治力度，坚决防止重特大事故发生。加强食品药品安全监管，保障人民群众“舌尖上的安全”。

市委市政府确定，今年为人民群众办好十二件实事：一是开通运营公交自行车1万辆，二是新建城市人行过街天桥或地下通道10座，三是新建城市公共停车场10座，四是建成区建设100座水冲式公厕，五是新建和改造提升10个便民市场，六是新建80个农村老年人日间照料中心，七是组织开展千场大戏、万场电影文化惠民活动，八是改造老旧住宅小区346万平方米，九是推进公共安全监控全覆盖，十是推进公共场所无线网络全覆盖，十一是综合整治小街小巷50条，十二是通过易地搬迁改善1．3万贫困人口的居住环境，通过教育助学资助1万名贫困家庭子女完成学业。

各位代表，群众的幸福感就是党和政府的成就感。我们要以为人民群众办实事为载体，积极顺应人民群众的期盼，生动彰显为民利民便民的执政理念，让广大人民群众在共建共享中不断增强改革发展的获得感和认同感。

（八）加强政府自身建设，着力提高行政效能。加快政府职能和作风转变，努力以政府的勤奋指数，提升城市的繁荣指数和人民的幸福指数。

不断深化行政改革。以行政权力瘦身为市场主体强身，及时承接国家和省取消下放的行政审批事项，抓好权力清单和责任清单动态管理。全面清理规范行政审批中介服务，推进行业协会、商会与行政机关脱钩改革，加强事中事后监管。完善政务服务和公共资源交易平台。推进电子政务建设，建成全市政务服务一张网。

大力推进依法行政。依法全面履行政府职能，加快建设法治政府。加强政府立法和规范性文件监督管理。做好行政复议应诉工作，启动“七五”普法，强化法律援助。健全完善重大行政决策公众参与、专家论证、风险评估等制度，逐步建立各级政府及其工作部门法律顾问制度。自觉接受人大法律监督、政协民主监督和社会舆论监督，认真听取民主党派、工商联和无党派人士的意见建议，充分发挥工青妇等人民团体的桥梁纽带作用。

持续转变工作作风。扎实开展“两学一做”学习教育，巩固“三严三实”专题教育、学习讨论落实活动和“冬季行动”的成果。严守政治纪律和政治规矩，增强政治意识、大局意识、核心意识、看齐意识，强化责任担当。筹划开展“发展环境优化年”活动，全面落实优化投资环境行动计划，把马上就办、真抓实干的作风固化下来、延续下去、形成常态。完善12345政府服务热线三级运行体系。积极稳妥开展政府绩效第三方评估试点工作。建立激励机制和容错纠错机制，对不作为、乱作为以及懒政、怠政干部严肃责任追究和问责处理。把督促检查贯穿于政府工作的全过程，完善“一报告、三清单”督查模式，打通政策落实中的“中梗阻”和“最先最后一公里”。

全面加强廉政建设。严格执行廉洁自律准则和党的纪律处分条例，切实履行“两个责任”和“一岗双责”，深入开展政府系统党风廉政建设和反腐败斗争。加强行政监察和审计监督，对容易滋生腐败和权力寻租的重点领域和关键环节严格监督。坚持不懈落实中央八项规定和国务院“约法三章”，驰而不息狠刹“四风”，厉行勤俭节约，反对铺张浪费，严控“三公”经费，切实做到为民、务实、清廉。

各位代表，宏伟蓝图已经绘就，壮丽前景催人奋进，神圣的使命激励我们勇往直前，人民的期望不容我们丝毫懈怠。让我们紧密团结在以习近平同志为总书记的党中央周围，在市委的坚强领导下，万众一心，开拓奋进，为夺取全面建成小康社会决胜阶段的伟大胜利，加快建设美丽大同、富裕大同、幸福大同而努力奋斗！
